# Vindication, virtue, and vitriol: A study of online engagement and abuse toward British MPs during the COVID-19 pandemic

**DOI:** 10.1007/s42001-020-00090-9

**Published:** 2020-11-17

**Authors:** Tracie Farrell, Genevieve Gorrell, Kalina Bontcheva

**Affiliations:** University of Sheffield, Sheffield, UK

**Keywords:** Online hate, Abusive speech, Natural language processing, Politics, COVID-19, Twitter

## Abstract

COVID-19 has given rise to a lot of malicious content online, including hate speech, online abuse, and misinformation. British MPs have also received abuse and hate on social media during this time. To understand and contextualise the level of abuse MPs receive, we consider how ministers use social media to communicate about the pandemic, and the citizen engagement that this generates. The focus of the paper is on a large-scale, mixed-methods study of abusive and antagonistic responses to UK politicians on Twitter, during the pandemic from early February to late May 2020. We find that pressing subjects such as financial concerns attract high levels of engagement, but not necessarily abusive dialogue. Rather, criticising authorities appears to attract higher levels of abuse during this period of the pandemic. In addition, communicating about subjects like racism and inequality may result in accusations of virtue signalling or pandering by some users. This work contributes to the wider understanding of abusive language online, in particular that which is directed at public officials.

## Introduction

Social media can offer a “temperature check” on which topics and issues are trending for certain cross-sections of the public, and how they feel about them [21]. This temperature has run high during the COVID-19 pandemic, with a number of incendiary and misleading claims [16], as well as hateful and abusive content [68] appearing online. This content can interfere with both government and public responses to the pandemic.

A recent survey of coronavirus conspiracy beliefs in England, for example, demonstrated that belief in conspiracy was associated with lower compliance with government guidelines. Moreover, the authors found that 1 in 5 of their participants had a strong endorsement of conspiracy thinking [25], indicating that this is not just a fringe issue. Online verbal abuse contributes as well, being both cause and consequence of misinformation: the quality of information and debate is damaged as certain voices are silenced/driven out of the space,^1^ and escalation leads to angry and aggressive expressions [60]. Understanding the interplay between malicious online content and the public’s relationships with authorities during a health crisis is necessary for an effective response to the COVID-19 pandemic.

### Scope

This work charts Twitter abuse in replies to UK MPs from before the start of the pandemic in the UK, in early February, until late May 2020, to plot the health of relationships of UK citizens with their elected representatives through the unprecedented challenges of the COVID-19 pandemic up to this point. We consider reactions to different individuals and members of different political parties, and how they interact with events relating to the virus. We review the dominant hashtags on Twitter as the country moves through different phases, as well as some dominant conspiracy theories. For these data, we show trends in abuse levels, for MPs overall as well as for particular individuals and for parties. We also compare prevalence of conspiracy theories, and contextualise them against other popular topics/concerns on Twitter.

In addition to our quantitative analysis, we present an in-depth qualitative analysis on tweets that had both a high amount and high percentage of abusive responses in our data. We use a set of qualitative codes derived from the literature on how authorities make use of social media during crisis (and health crisis in particular). Referring to this as the social media activities of the MP who authored a tweet, we label each tweet according to its potential agenda (e.g., reaching out to constituents, communicating official information). This allows us to see the distribution of media activities across different parties and genders, for example. We then developed inductive codes for the COVID-19 topic (e.g., policy, communication and leadership) and potential controversial subject of the tweet (e.g., that the tweet makes criticisms of COVID-related policy, or discusses a touchy subject like Brexit or Inequality). We also noted any attached URLs or images for reference and listed the abusive words found in each reply to the tweet. Finally, we prepared an analysis of those labels by gender, party, sexual orientation, and ethnicity. In our analysis, we consider how social dimensions of antagonistic political discourse in the UK (ideology, political authority, and affect), which have been visible in other recent key moments (such as Brexit and successive general elections), influence the civility of discourse during COVID-19.

### Contribution

This study aims to answer the following research questions:

1. How has the context of COVID-19 impacted the typical patterns that have been observed in previous work about hateful and abusive language toward UK MPs?
2. How do the social dimensions that have impacted political discourse on Brexit and successive general elections appear to impact how social media activities are perceived during the COVID-19 pandemic?
3. Which social media activities of UK MPs during the COVID-19 pandemic receive the most abusive replies? How can we contextualise these results?

The contribution of this study is to understand both the content and context of abusive and hateful communication, particularly toward governments and authorities during a health crisis. Our focus, UK MPs, adds to a growing longitudinal body of work that analyses online abuse at many key moments in British politics from 2015 to the present [6, 28, 34, 75].

In the following sections, we begin with a description of the current context and a brief summary of related work. We then outline our methodology in detail before progressing onto findings. Finally, we summarise and conclude our manuscript with some suggestions for future work.

## Context of COVID-19

The dangers and perceived risks of COVID-19 have fluctuated during the pandemic as a result of emerging knowledge. This feature of the pandemic creates an environment of uncertainty and ambivalence that feeds malicious content on social media.

Early epidemiological studies of COVID-19 [44, 77] implicated certain risk factors, such as age, gender, and pre-existing conditions, which may impact transmission and severity of illness. As the pandemic progressed, researchers began to understand more about asymptomatic transmission [3, 18, 52], discovering that there may be many more cases of COVID-19 than once realised. This led some researchers to suggest that the morbidity rate of COVID-19 is lower than initially presumed [23], though these data are difficult to calculate.^2^

Over time, some communities were discovered to be at a greater vulnerability. Research on health care professionals who contracted COVID-19, for example, indicated that the most seriously ill individuals had multiple exposures to the virus, primarily at work [12], as well as longer duration of (sometimes unprotected) exposure to the virus [37]. As more information emerged about disproportionate cases of COVID-19 and poor health outcomes in Black and Asian communities in England, researchers began to also investigate early warning signs that some social or ethnic communities were more vulnerable than others [41, 53]. This too has led to challenging debates about social welfare, racism, and healthcare during the pandemic. Knowledge about the virus and its transmission continues to evolve.

Tensions between competing political and social interests during times of uncertainly can lead to an increase in antagonistic discourse, in particular, if the public do not feel that threats can be managed [65]. This leaves the door open for antagonistic groups to spread hate and malicious content even into mainstream communities [68].

## Related work

Organisations use social media during crisis events to correct rumours, prevent crisis escalation, provide facts or information, transmit proactiveness toward resolving the situation, and to communicate directly with members of the public (without temporal or geographic constraints) [20]. Not using social media to address a crisis can incur reputational damage for the organisation [17].^3^

Twitter and other forms of social media are popular tools used by organisations and governments to communicate with citizens during crisis events [55]. The focus for the literature below is to briefly review how governments and authorities use such tools to communicate about health crises, particularly in the UK, and to explore how malicious content and abuse has been examined previously within this context.

### Public use of social media in a health crisis

Before we address how politicians use social media in a health crisis, it is worth examining perspectives of the public and what they expect from politicians when emergencies like COVID-19 arise. Evidence indicates that the public expect a swift, transparent response from the government to crisis [7, 55]. The public may also wish to engage with the government on its response. The greater the political interest of the user, the more likely they are to perceive and take advantage of the “connective affordances” that social media provides for politicians and their constituents to engage [40]. Third, and perhaps most importantly, during a health crisis, most citizens will be interested in government advice and support [46, 67]. For example, Vos and Buckner examined Tweets that were shared during H7N9 “Bird Flu” health crisis, and found that the majority of messages were about “collective sense-making responses” under conditions of uncertainty, rather than “efficacy responses” offering specific advice or information that would help the public to respond appropriately [70]. A similar pattern was observed in response to the 2016 Zika virus outbreak, with individuals using social media to form a personal risk assessment [35]. Llewellyn et al. [46] found that the public seeks advice from experts and that the informal character of online communication can interfere with the public’s ability to form good opinions about the expertise of individuals online, even public figures. If sense-making and risk-assessment are the top public tasks for which they seek information on social media, government messages that do not respond to this need may miss the mark.

### Politicians’ social media use in a health crisis

In their analysis of political communication on social media, Stieglitz and Dang-Xuan [63] show that politicians may use social media for communication and persuasion, to “meet” voters and engage them in discussion, and also to communicate policy or other important information to their constituents. Political analysis of US congress members on Twitter shows that self-promotion is also an activity in which politicians engage, using the opportunity to share personal information or stories, and present themselves and their platforms in a good light [27]. However, this is not always true. Studies from the Swedish electoral context showed that Swedish politicians did not use Twitter to engage with voters, but rather to provide information to them [43]. In the UK, because internal party campaigns are based on individual candidates, politicians in the UK share some media behaviours with their counterparts in the US, where individual voter appeal is critical to campaign success [45].

#### Dimensions of political discourse in the UK

The COVID-19 pandemic is a novel political situation in which ministers must respond to the crisis, while continuing to function in their roles. Though the situation may be new, the dialogue around COVID-19 is influenced by existing social and political dimensions of British political discourse.

In their work documenting positions around the European Referendum, Andreouli et al. named three dimensions for understanding the emergent dialogue around Brexit [1], that we feel may be useful here. These are “political values, political authority, and the authority of affect”.

With regard to values, the authors reflect on how existing ideological themes impact how an issue is perceived and discussed, in particular, where classical dichotomies do not hold up. For example, while the left typically associates itself with anti-prejudice and tolerance, associating such qualities with voting to “remain” is inconsistent with other leftist ideas to be anti-establishment. The authors argue that this tension creates a “liminal hotspot” where cosmopolitanism and critiques of globalisation intersect.

We propose this same step, in light of the current crisis, to understand the dominant political and social themes that influence abuse toward UK MPS during COVID-19. We are already seeing evidence of potential areas of tension in the current pandemic, such as the needs of older^4^ and younger^5^ people, the reliance on science and perception of risk [11, 25], the division between the wealthy and the poor [72], and the experiences of the urban and rural [39, 49].

Second, Andreouli et al. [1] discuss the notion of political authority, and how the sovereignty of the UK within the EU becomes a backdrop for discourse on immigration during the European Referendum. During the COVID-19 pandemic, the sovereignty of local governments within the UK has been a consistent feature of debate, whether it involved avoiding beauty spots in Wales during lockdown,^6^ comparing Scotland’s success in handling the virus,^7^ or the differential impact on the economy in Northern Ireland.^8^ Media reports during the peak of the outbreak also indicated resistance toward lockdown,^9^,^10^. and wearing face-coverings.^11^,^12^ As we move toward the next phases of the crisis, conflicts about personal agency and choice are likely to play out at both individual and group levels in how the public respond to government guidance.

Finally, Andreouli et al. [1] discuss the role of affect in political discourse. They demonstrate how impassioned speech has become its own kind of credibility, in which the narrative, rather than being factual, is important. In September 2019, parties signed a pledge to use moderate language after a series of heated and antagonistic debates. This discussion highlights how ideology, values, and cultural awareness influence language perception.^13^

One of the goals of this research is to analyse topics of discussion and responses within these three dimensions (see “Dimensions of political discourse during COVID-19 (RQ2)”). This will allow us to contextualise the public’s antagonistic responses to UK MPs on Twitter during COVID-19 thus far.

#### Hate and abuse of British MPs online

In the UK, where legal frameworks tend to evolve, hate speech was defined through several legal statutes, including the Public Order Act of 1986, clarifying the groups in need of protection.^14^ However, more generally, hate speech is communication to marginalised groups or communities (and their sympathisers) that they are not welcome or wanted [71]. Crucially, hate speech is associated with power [54].

Governments and politicians have communicated hateful messages, for example, through language about Romani people in Europe [59] or Mexican and other Latin immigrants in the United States [14, 42], all of which experience discrimination in their host countries. Governments have contributed to hate through politicising tribal identity in sub-Saharan countries like Kenya and Rwanda [24, 61], and how they shape debates about free speech [56] or migration [66] more generally. Politicians, therefore, can both be the targets of hate (as members of protected groups) and the perpetrators (as public authorities whose words matter). Governments can also antagonise the public. In the context of COVID-19, the ways in which politicians are communicating about potentially volatile issues, such as re-opening the economy or avoiding social protest, add to the overall “health” of the discourse, or diminish it.

There is a considerable amount of historical data on the prevalence of online abuse directed at British MPs, particularly on Twitter. Previous work [6, 28, 34, 75] has shown rising levels of hostility towards UK politicians on Twitter, particularly in the context of divisive issues, such as Brexit or inequality. Partisan operators have been implicated in fanning the flames with malicious content, such as misinformation or troll accounts [31].

In their 2017 and 2020 papers, Ward and McLoughlin examined online abuse received by British MPs from November 14, 2016 to January 28, 2017 [51, 74]. The major findings from this work were that the amount of online hate (rather than language that can be described as “abusive” or uncivil) is relatively low and, as such, men receive more online abuse than women. The authors also showed that increased name recognition and popularity have a positive relationship with levels of abuse. Crucially, however, the authors note that women and those from a minority background are more likely to receive abusive replies that can be classified as hate speech. Abuse toward specific parties was difficult to distinguish, as levels of abuse may be influenced by one party member who attracts a significant proportion of abuse. When controlling for this, the authors found that less visible MPs had a very small percentage of hate and abuse. This means that women MPs with visibility disproportionately attract hate speech, as do men with visibility other forms of abusive language. This work prompted questions about what visibility means for people of different genders and backgrounds. Southern and Harmer [62] conducted a deeper content analysis on tweets received by MPs during a period, and found that while men received more incivility in terms of numbers of replies, women were more likely to receive an uncivil reply. Women were more likely to be stereotyped by identity (men by party) and to be questioned in their position as an MP.

Gorrell et al. [30] extended this work to define four visibility factors that appear to influence the amount of abuse the UK MPs receive online:

- Prominence: Individuals in the public eye will receive more abuse;
- Event surges: Events leads to spikes in abuse (such as participation in an event or political activities);
- Engagement: Expressing strong opinions on social media can result in more personal abuse;
- Identity: Gender, ethnicity, and other personal factors impact which opinions one is allowed to hold and express without receiving abuse.

Gorrell et al. also note that the impacts or consequences of abusive language are not manifesting in the same ways for male and female MPs, or MPs with intersectional identities of race and gender. Where some abuse is distressing, other abuse is personal and threatening, and limits women’s participation in the public office [19, 28, 57].

From this review, a picture emerges of the precipitating activities, mediating factors and dimensions of online abuse toward UK MPs during COVID-19, which can be interrogated through our large-scale study.

## Methodology

In this work, we apply a combination of computational and social science methods to evaluate abuse toward UK MPs on Twitter. We utilise a large tweet collection on which natural language processing analysis has been performed to identify abusive language. This methodology is presented in detail by Gorrell et al. [28] and summarised here. We then follow Braun and Clarke’s [10] process of thematic analysis on a subset of tweets that received 8% or more of abusive replies, in which at least 20 abusive replies were received. This analysis is described in more detail below.

### Corpus

The corpus was created by collecting tweets in real time using Twitter’s streaming API. We used the API to follow the accounts of UK MPs—this means that we collected all the tweets sent by each current MP, any replies to those tweets, and any retweets either made by the MP or of the MP’s own tweets. Note that this approach does not collect all tweets which an individual would see in their timeline, as it does not include those in which they are just mentioned. However, “direct replies” are included. We took this approach as the analysis results are more reliable due to the fact that replies are directed at the politician who authored the tweet, and thus, any abusive language is more likely to be directed at them. Data were of a low enough volume not to be constrained by Twitter rate limits.

The study spans February 7th until May 25th 2020 inclusive, and discusses Twitter replies to currently serving MPs that have active Twitter accounts (574 MPs in total). Table 1 gives the overall statistics for the corpus.

**Table 1:** Corpus statistics

Original	Retweet	Reply	ReplyTo	Abusive	% Abuse
107,209	187,586	56,706	4,726,070	179,493	3.798

Figures give number of original tweets authored by MPs, number of retweets authored by them, number of replies written by them, number of replies received by them, number of abusive replies received by them, and abusive replies received as a percentage of all replies received

### Rule-based identification of abusive language

A rule-based approach was used to detect abusive language, as in previous work [30]. An extensive vocabulary list of slurs (e.g., “idiot”), offensive words such as the “f” word and potentially sensitive identity markers, such as “lesbian” or “Muslim”, forms the basis of the approach. The slur list contained 1081 abusive terms or short phrases in British and American English, comprising mostly an extensive collection of insults, racist, and homophobic slurs, as well as terms that denigrate a person’s appearance or intelligence, gathered from sources that include http://hatebase.org and Farrell et al. [22]. 131 offensive words were used, along with 451 sensitive words. “Bleeped” versions such as “f**k” are also included.

On top of these word lists, 53 rules are layered, specifying how they may be combined to form an abusive utterance as described above, and including further specifications such as how to mark quoted abuse, how to type abuse as sexist or racist, including more complex cases such as “stupid Jew hater”, and what phrases to veto, for example “polish a turd” and “witch hunt”. Making the approach more precise as to target (whether the abuse is aimed at the politician being replied to or some third party) was achieved by rules based on pronoun co-occurrence. Where people make a lot of derogatory comments about a third party in their replies to a politician; however, for example racist remarks about others, there may be targeting errors leading to false positives.

Data from Kaggle’s 2012 challenge, “Detecting Insults in Social Commentary” [5], were used to evaluate the success of the approach, this being in keeping with our definition of abuse (many more recent corpora define this differently, e.g., “toxicity”, as in the Jigsaw corpus [9], is much broader). Our approach was shown to have an accuracy of 80%, and a precision/recall/F1 of 0.72/0.47/0.57. This precision is considered sufficient for empirical work (being greater than 0.7 [58]). However, there is a long tail of linguistically more complex abuse that is hard to identify with sufficient precision, and therefore, recall is low. Being a hard problem, this is not dissimilar to the state of the art on cross-domain data [76], but the main reason for this choice of approach lies in the critical importance of unbiased classification for this type of work—naively applied machine learning approaches result more false positives for, e.g., ethnic minorities or women [8, 13, 36]. Future work will explore promising but quite preliminary work in fair abuse classification [26, 38]. The resulting system is publicly available at https://cloud.gate.ac.uk/shopfront/displayItem/gate-hate.

The method for detecting COVID-19-related tweets is based on a list of related terms. This means that tweets that are implicitly about the epidemic but use no explicit COVID terms, for example, “@BorisJohnson you need to act now,” are not flagged.

### Thematic analysis

To understand more about the kinds of tweets attracting high levels of abuse, we considered several approaches for ranking them. Ranking tweets by the most replies will surface prominent individuals, but perhaps not always polarising individuals or viewpoints. We decided on an initial criterion that a tweet had to have received 8% or more abusive replies, which is nearly twice the average level of abuse noted by [29, 34, 75]. In addition, to filter out tweets that were attracting just a handful of abusive replies, we examined tweets that received at least 20 abusive replies. We had 191 tweets in this sample.

All tweets were first examined and coded openly, as suggested by Braun and Clarke [10], to see which patterns emerge. Contentious subjects (such as Brexit, racism, and even Jeremy Corbyn), as well as potential media agendas (such as reaching out to under-valued communities, or making criticisms of the party in government) emerged from this analysis. We then compared open codes to themes from the literature regarding social media activities that politicians may undertake during a health crisis, as well as ideological themes and existing priors that may be influencing how UK MPs are perceived. We created a final set of categories through the processes of reduction and comparison across the codes. We then re-coded the data according to the final annotation scheme in Table 2. While the sample is small, which reduces potential for generalisation, this qualitative analysis is meant to highlight new and nuanced relationships between what is being said and how the public is responding.

**Table 2:** Media categories that express MPs’ activities on Twitter, to which the MP received abusive replies

Media category	No. tweets	Description	Examples
Defending	14	MPs responding to critiques of oneself or others	*Replying to Keir Starmer: A low blow and misjudged at this time. Yes we need everyone to have PPE but the right use of It is also needed.*- Stuart Anderson
Defending (e)	3	Similar to the above, but with “escalation indicators” (described below)	*For God’s sake. The man nearly died. He is going to Chequers away from the glare of publicity to recuperate and be with his partner who is due to give birth in a matter of weeks. Get a life - idiot keyboard warrior! #HardHearted* - Andrea Jenkyns
Direct Rebuke	3	MPs critiquing someone who is not directly an authority	*No celebrations today. Only relief that the disastrous Corbyn era is over and hope that we can turn the page on what it did to Labour. Congratulations to (Kier Starmer) and (Angela Rayner) on their elections as Leader and Deputy Leader.* - Pat McFadden
Direct Rebuke (e)	3	Similar to the above, but with escalation indicators	*Not that I should be surprised by the lazy left but interesting how work-shy socialist and nationalist MPs tried to keep the remote Parliament going beyond 2 June.* - Henry Smith
Direct Rebuke of Authorities	60	MPs critiquing people in power, in particular coming from opposition parties	*It is irresponsible and short-sighted from the government to rule out extending the post-Brexit transition period. We should be taking action now to provide certainty for business in the face of this global economic challenge.* - Lisa Nandy
Direct Rebuke of Authorities (e)	70	Similar to the above, but with escalation indicators	*Boris Johnson boasting about shaking hands with coronavirus patients. You could not make it up. Britain is about to learn the hard way this is not the man to lead us in a crisis.* - David Lammy
Engage Voters	47	MPs reaching out to potential new voters and speaking to core voters	*If there’s one clip to watch from #Brits2020 last night, it’s this. (Santan Dave) speaking truth to power.* Hero - Zarah Sultana
Escalation	9	MPs making statements that just agitate	*How many Johnson children are there now?* - Barry Sheerman
Events	12	The MP’s tweet relates back to an event that preceded the Tweet or a clear pattern of behaviour	*NEWS: My Telegraph article on the next stage of our #coronavirus plan: We must all do everything in our power to protect lives.* - Matt Hancock
Information	4	MPs providing information without commentary	*Data sources and maps here:* - Richard Burgon
Proactive	24	MPs relating a sense of doing something about the problem	*Dear @patel4witham please do the right thing and release the women in Yarlswood now.* - Jess Phillips
Unclear	4	Tweet could not be annotated or belongs to no clear category	*Quite the change in rhetoric from the days when Welsh Government were encouraging people to come to Wales and drive their* $4 \times 4's$ *right onto the beaches.* - Stephen Crabb
Grand Total	190		

Descriptions and examples included

In addition to these codes, we also examined the topics MPs referred to in their posts and grouped them inductively into categories of topics that are considered controversial in UK politics. These were: home rule/nationalism with respect to Northern Ireland, Scotland or Wales; inequality; and Brexit, alongside specific individuals and the ways in which they communicate. Of course, the topic of COVID-19 itself—the government response and the impacts—was a primary topic category, as well. Following the distribution of these codes and topics in our sample, we used descriptive statistics and further qualitative analysis to expand on trends and observations uncovered through our computational approaches.

In consideration of rigour, we have taken several steps to adjust for having used one annotator for the qualitative analysis. Barbour has suggested focusing on alternative explanations in analysis, rather than a potentially superficial measure of inter-rater agreement through multiple coders [4]. In addition, we provide a full justification of the coding scheme against each tweet in the sample, available at the URL noted in “Availability of data and material”, so that other researchers can interrogate and interpret our findings accordingly.

In the following section, we present our findings, which are organised by research question and which include both the quantitative and qualitative data analysis that answer that particular research question.

For RQ1, we rely primarily on our literature review of trends and high-level analysis of abuse toward British MPs. We compare this with our findings from the COVID-19 period which we studied. For RQ2, we consider how our findings fit the dimensions noted by Andreouli et al. [1] as impacting contemporary British political discourse: ideology, authority, and affect. We explore the four factors that contribute to how these dimensions are perceived, such as prominence, specific events, engagement habits, and features of identity [30]. Finally, for RQ3, we present our qualitative analysis on the social media activities of UK MPs during COVID-19 thus far. We contextualise the abusive responses which they receive for these activities, given the dimensions and contributing factors that may play a role.

## Findings: general trends and comparisons (RQ1)

Our first research question asked: How has the context of COVID-19 impacted the typical patterns that have been observed in previous work about hateful and abusive language toward UK MPs? To answer this question, we begin with a review of the time period studied, namely February 7th until May 25th 2020 inclusive, placing it in historical context.

Gorrell et al. [33] use the same (cautious) abuse counting methodology as we use here to show that aside from a blip around the 2015 general election, abuse toward MPs on Twitter has been tending to rise from a minimum of 2% of replies in 2015, peaking mid-2019 at over 5% with a smaller peak of around 4.5% around the 2019 general election. After the election, however, abuse toward MPs fell to around 3.5%.

**Fig. 1:**
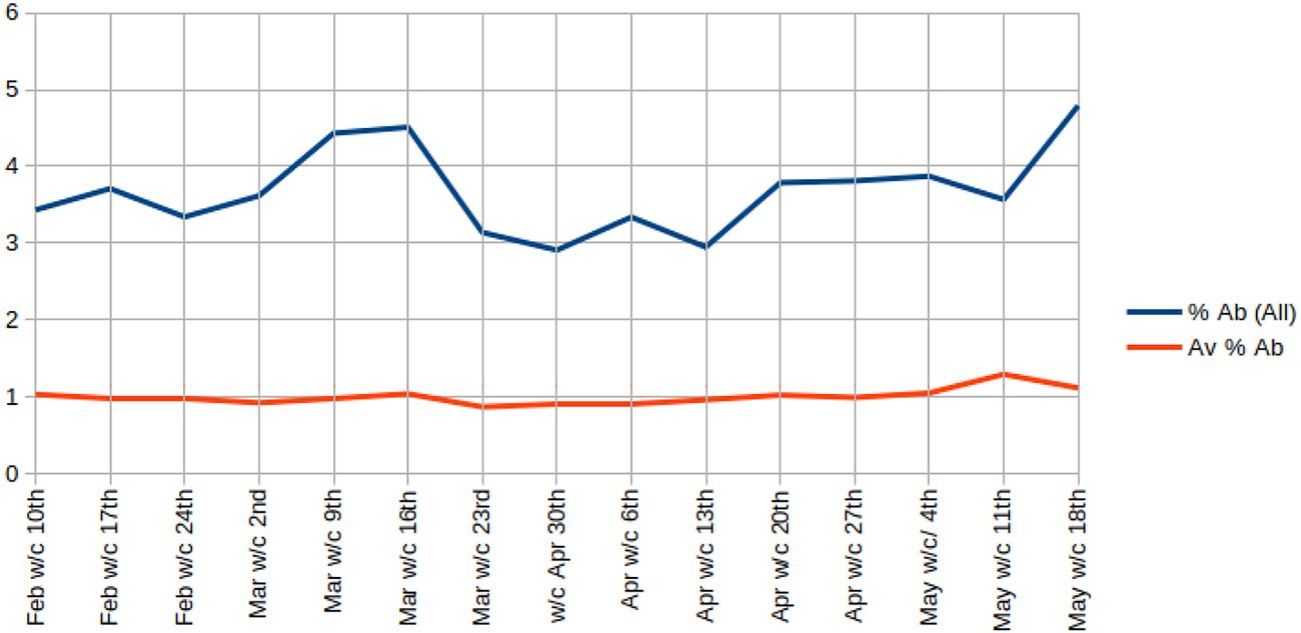
Abuse percentage received by all MPs, macro- (red) and micro- (blue) average, per week

In the timeline in Fig. 1, we zoom in on the study period, and show abuse levels overall, toward all MPs, on a per-week basis since mid-February. This timeline shows a rise in abuse, back up to over 4% around the time of the introduction of social distancing, before dipping, and then gradually beginning to rise again later in the study period. We see that the macro-average abuse level (red line, averaged across MPs rather than across pooled counts, [48] p. 577) remains relatively steady, suggesting that this fluctuation is confined to a small number of high profile politicians (therefore being more evident in the micro-averaged blue line).

**Fig. 2:**
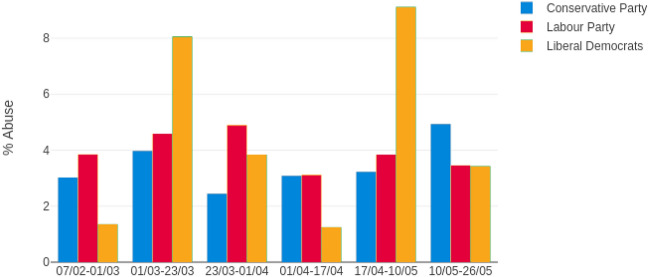
Abusive replies as a percentage of all replies received, micro-average, split by party, and time period

Figure 2 shows abuse received as a percentage of all replies received by MPs, for six distinct time periods discussed in more detail below. We see that, on the whole, response to the conservative party has been favourable. The exception is after May 10th, when the negative response to Dominic Cummings’ decision to travel north with COVID-19 symptoms came to the fore. Responses to Liberal Democrat MPs are more erratic due to their lower number. In previous studies, we have found conservatives receiving higher abuse levels, yet here we see Labour politicians receiving more abuse in most periods. This was in evidence even in February, so precedes the pandemic, although Twitter has tended to be left-leaning in the UK [32]. It remains to be seen if this is the beginning of a swing to the right or if it is specific to the times, e.g., arising from a desire to trust authority during times of crisis [69]. It may even be related to the presence of polarising characters, for example Mr. Corbyn, in comparison with new Labour leader Keir Starmer.

There is a significant negative correlation between receiving a high level of COVID-related attention and receiving abuse ($-0.52, p<0.001$, Feb 7th to May 25th, Spearman’s PMCC). We see this clearly in prominent government figures below, who are receiving the lion’s share of the COVID-19 attention and lower levels of abuse than seen for them in pre-COVID periods [30, 34]. However, the correlation is significant across the sample of all MPs. The reaction of the public to the Conservative party and the government’s actions during COVID-19 may be related to the conditions of a public health crisis as discussed in [46, 67], in which citizens may feel more motivated to trust authorities, although it may also follow from the crisis engaging a different group of people than usually respond to politicians on Twitter. Future work will have to examine the strength of this finding.

With a view to separating out different groups of Twitter users, we tracked hashtags relating to dominant pro- vs anti-lockdown perspectives, as well as issues of concern; namely conspiracies and misinformation, and racism in conjunction with the pandemic. Pro- and anti-lockdown hashtags were easily acquired, being dominant hashtags appearing in the dataset. They were then extended with minor linguistic variants. This report^15^ from Moonshot CVE was used as a guide to the overall conspiracy landscape within COVID-19. They provide some hashtags, and variants were then acquired, again, from looking down the list of hashtags appearing in the dataset for other variants, and including linguistic variations. The areas which they highlight are anti-Chinese feeling/conspiracy theory, theories that link the virus to a Jewish plot, theories that link the virus to an American plot, and generic “deep state” and 5g-based theories and general theories that the virus is a plot or hoax. Table 3 shows substantial evidence of ill-feeling toward China.

In our analysis, MPs using Chinese data or referencing the Chinese government’s response to COVID-19 in a positive context appear to attract abuse. One example, in terms of receiving a high percentage of abuse as well as a notable degree of attention, was the one below from Richard Burgon.

https://twitter.com/RichardBurgon/status/1244043022297370626 (17% abuse or 6% of all abuse sent to MPs in March post-lockdown):

This is a Trump-style attempt to divert blame from the UK government’s failures.

A World Health Organization report says China “rolled out perhaps the most ambitious, agile & aggressive disease containment effort in history”

We haven’t even sorted out enough tests for NHS staff

China’s record of human rights or transparency is often provided as evidence of argument against such tweets. However, mixed in with this are also a number of Sinophobic comments about China having “caused” or “started” the virus, for which, at present, there is no reliable scientific evidence. It is not clear how potentially useful critiques of the Chinese government may be discussed without also provoking more sinister, racist commentary.

Classic conspiracy theories are in evidence, but numbers of mentions are low (though note that most of the 183 mentions of “NWO” (“new world order”) are now COVID-19-related, suggesting opportunistic incorporation of COVID-19 into existing mythologies). There is considerable evidence of some Twitter users not believing in the virus, and numbers of mentions to this effect are within one order of magnitude of the popular “stay home save lives”. Yet, all are surpassed by the theme of economic support for those not in established employment (“#newstarterfurlough”).

**Table 3:** Mention count of viewpoint-related hashtags, in all replies to MPs, Feb 7th to May 25th inclusive

Search terms (in all replies to MPs, not case-sensitive)	# tweets	# abusive	% abusive
“#endthelockdown”	5,506	272	4.940
“#newstarterfurlough” OR “#newstarterjustice”	55,593	243	0.437
“#stayhomesavelives”	20,538	1222	5.950
“#coronahoax” OR “#hoaxvirus” OR “#fakevirus”	415	61	14.699
“#coronabollocks”	250	19	7.600
“#plandemic” OR “#scamdemic” OR “#fakepandemic”	1178	64	5.433
“#filmyourhospital” OR “#emptyhospitals”	59	5	8.475
“#ccpvirus” OR “#chinaliedpeopledied”	2463	38	1.543
“#chinesevirus”	2208	119	5.389
“#NukeChina” OR “#DeathtoChina” OR “#DestroyChina”	31	2	6.452
“#GatesVirus” OR “#CIAVirus” OR “#AmericaVirus”	69	2	2.899
“#5gcoronavirus”	53	1	1.877

Some further variants of the terms given, including non-hashtag mentions in text, are also included but not listed here for brevity; see Gorrell et al. [33] for a more complete description

So across the board, COVID-19 has not led to higher proportions of abuse on Twitter for MPs compared with the high levels of abuse directed at them in 2019. However, these findings might be partially explained by varying degrees of engagement by different societal groups, in addition to events affecting attitudes to authority. As we will see in “Political authority”, the comparatively positive response to Boris Johnson might be explained by more people replying to him than would normally do so; this extra attention was not abusive. The lower levels of abuse received by MPs who receive more tweets mentioning COVID-19 might also be explained by different people replying to politicians than usually would. A particularly striking illustration of this comes from tweets to MPs using the hashtag #newstarterfurlough and variants. People who had recently started a new job “fell through the cracks” for financial support from the government. With 56,000 tweets to MPs using #newstarterfurlough and variants (compared with only 21,000 using #stayhomesavelives and variants), #newstarterfurlough is the dominant hashtag campaign of the period. Given that those individuals are in an unfortunate position, it is all the more surprising to find that only 0.4% of those tweets contained abuse, as shown in Table 3. A possible explanation is that the “new starters” are a broader, and more polite, cross-section of society than people who usually reply to politicians on Twitter.

In contrast, tweets containing #stayhomesavelives and variants contained 6.0% abuse. Tweets containing hashtags refuting the very existence of the virus, for example #scamdemic and #hoaxvirus, contained 7.8% abuse. Tweets describing COVID-19 as “Chinese”, e.g., containing #chinesevirus, contained 5.4%. Tweets found containing anti-lockdown hashtags contained 4.9% abuse.

## Dimensions of political discourse during COVID-19 (RQ2)

Our second research question asked what are the societal dimensions that appear to impact how media activities are perceived during the COVID-19 pandemic and how does this compare with those that impacted Brexit or recent general elections? For this question, we drew on previous work described in “Related work”, more specifically in “Politicians' social media use in a health crisis” on levels of abuse toward UK MPs. We then examined the time period covered by our study in the context of three dimensions drawn from Andreouli et al. [1]: political authority, ideology, and affect. We use the four factors from Gorrell et al. [30] to help further describe these dimensions in terms of: prominence, event surges, engagement, and identity.

### Political authority

As mentioned previously, Brexit created a notion of political authority that presented sovereignty of the UK on one side or community with Europe on the other [1]. During the past three UK elections, partisanship has led to a splintering of political authority and an erosion of trust [31]. During the COVID-19 pandemic, we can see a similar effect, for example, the wearing of face-coverings as a personal versus social choice,^16^ and participation in protest versus public health.^17^

Increased name recognition and popularity have also been associated with higher levels of abuse in both the cases of Brexit and General Elections [30, 34, 51, 74]. Our COVID-19 data show similar trends.

**Fig. 3:**
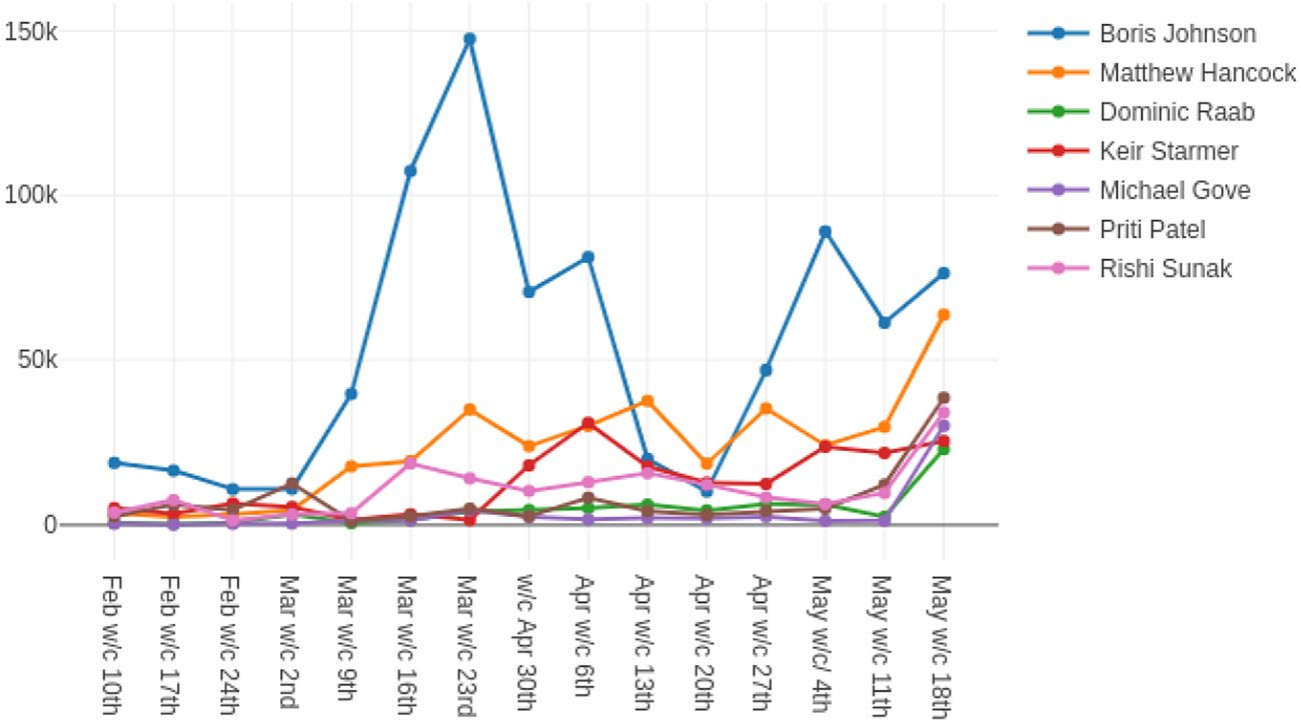
Number of replies received by relevant ministers and opposition leader Keir Starmer per week from February 7th to May 25th 2020 inclusive. Central peak relates to Boris Johnson’s illness with COVID-19; late uptick arises from Dominic Cummings’ controversial trip north

Figure 3 shows number of replies to prominent politicians since early February, and shows that, for the most part, attention during COVID-19 has focused on Boris Johnson. He received a large peak in Twitter attention on March 27th. 58,286 replies were received in response to his tweet, announcing that he had COVID-19. Abuse was found in 2.3% of these replies. This is low for a prominent minister as we may discern from Fig. 1, indicating a generally supportive response to the prime minister’s illness. Further peaks on Mr Johnson’s timeline correspond to the dates on which he was admitted to intensive care (April 6th), left hospital to recuperate at Chequers (April 12th), and began to ease the lockdown (May 10th). The late burst of attention on other politicians arises from several tweets by ministers in support of Dominic Cummings, the senior government advisor who chose to travel north to his parents’ home in the early stages of his illness with COVID-19.

However, one tweet receiving a high level of abuse regarded the very first video address made by Boris Johnson in response to the pandemic:

https://twitter.com/BorisJohnson/status/1238365263764041728 (9% of replies were abusive; tweet received 3% of all abuse to MPs in the period. It also includes a video statement.)

This country will get through this epidemic, just as it has got through many tougher experiences before.

For those who trust in Boris Johnson’s leadership and who like the way he communicates, this tweet may have provided some comfort. A review of replies to this tweet shows that supporters tweeted messages of appreciation and hope in response. For those who do not trust him and who believe that he should have acted sooner, this tweet was perceived as a provocation. Several replies are critical but not abusive point to official sources of information from elsewhere in Europe, or make advisement to the public about staying home and avoiding non-essential journeys.

In this sense, COVID-19 lends its own political authority to some arguments. However, invoking COVID-19 as a member of the opposition, especially in the context of persistent debates, is often met with accusations of “playing party politics”. The following tweet by David Lammy received 16% abusive replies, representing 1% of all abuse sent to MPs in the March pre-lockdown period:

https://twitter.com/DavidLammy/status/1239835712444391424

No more government time, energy or resources should be wasted on Brexit this year. Boris Johnson must ask for an extension to the transition period immediately. #COVID19 is a global emergency.

Several MPs made comments about the need to extend the Brexit transition period. All received a considerable amount of replies containing abusive language.

More generally, there is disagreement about the priorities of government during a crisis. For example, there was a considerable amount of abuse directed at Richard Burgon for a tweet in which he discussed the accomplishments of his work as shadow justice secretary.^18^ This was regarded by some critics as mistimed, given the PM’s health at the time, and received 17% abuse, constituting 2% of all abuse sent to MPs between April 1st and 16th inclusive.

In later sections, we will explore how the media activities of opposition parties are perceived by the public, in particular in connection with contentious subjects.

### Events

Events are somewhat in a category of their own, as an MP’s past actions and words become part of the public’s priors in understanding the position of that MP. To contextualise the level of attention to MPs and the abusive replies they received in connection with specific events, we review the events of the period, both in terms of who is receiving abusive replies and in the themes that are rising during the period as demonstrated by the appearance of certain hashtags.

#### Beginning of the pandemic

**Fig. 4:**
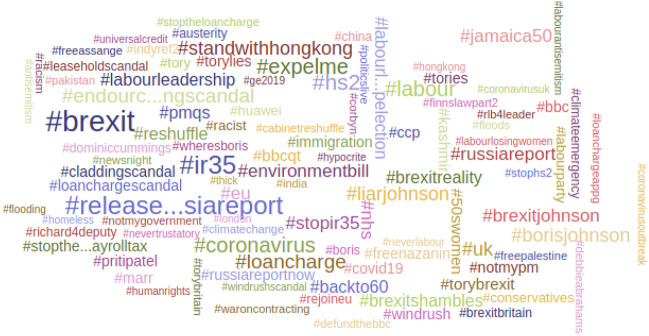
Top 100 hashtags in all replies sent to MPs—February 7–29 2020 inclusive

The hashtag cloud in Fig. 4 shows that Brexit remained the dominant topic in Twitter political discourse during February, with the epidemic not yet having arrived in the UK.

Table 4 gives a baseline for attention on MPs as we go into the pandemic, showing that aside from Boris Johnson, attention, and abuse, is high for Labour politicians. The column “Authored” refers to the number of tweets originally posted from that account that were not retweets or replies. “replyTo” refers to all of the replies received to the individual’s Twitter account in that period. The next column, “COV”, is the number of replies received to that account containing an explicit mention of COVID-19, with the following column representing the number of replies that verbal abuse was found in (“Abusive”). The last three columns present the data in a comparative fashion. First, we have the percentage of replies that the individual received that was abusive. Next, we have the percentage of replies that were COVID-related. The last column is the percentage of COVID-related replies to that individual, in comparison with all COVID-related replies received by all MPs.

**Table 4:** MPs with greatest number of replies from February 7 to February 29 2020 inclusive

Name	Authored	ReplyTo	COV	Abusive	% Ab	% COV	% Total COV
**Boris Johnson**	14	48,379	1,072	1695	3.504	2.216	37.773
*David Lammy*	89	47,368	73	2308	4.872	0.154	2.572
*Richard Burgon*	184	30,789	18	1556	5.054	0.058	0.634
*Jeremy Corbyn*	25	29,550	215	2400	8.122	0.728	7.576
*Rebecca Long-Bailey*	121	27,113	17	823	3.035	0.063	0.599
*Zarah Sultana*	96	18,630	12	677	3.634	0.064	0.423
*Debbie Abrahams*	96	18,186	45	822	4.520	0.247	1.586
*Keir Starmer*	97	15,455	26	360	2.329	0.168	0.916
*Lisa Nandy*	110	15,271	9	413	2.704	0.059	0.317
**Priti Patel**	9	13,664	45	345	2.525	0.329	1.586

Cell indicate party membership; bold for Conservative, italics for Labour

**Fig. 5:**
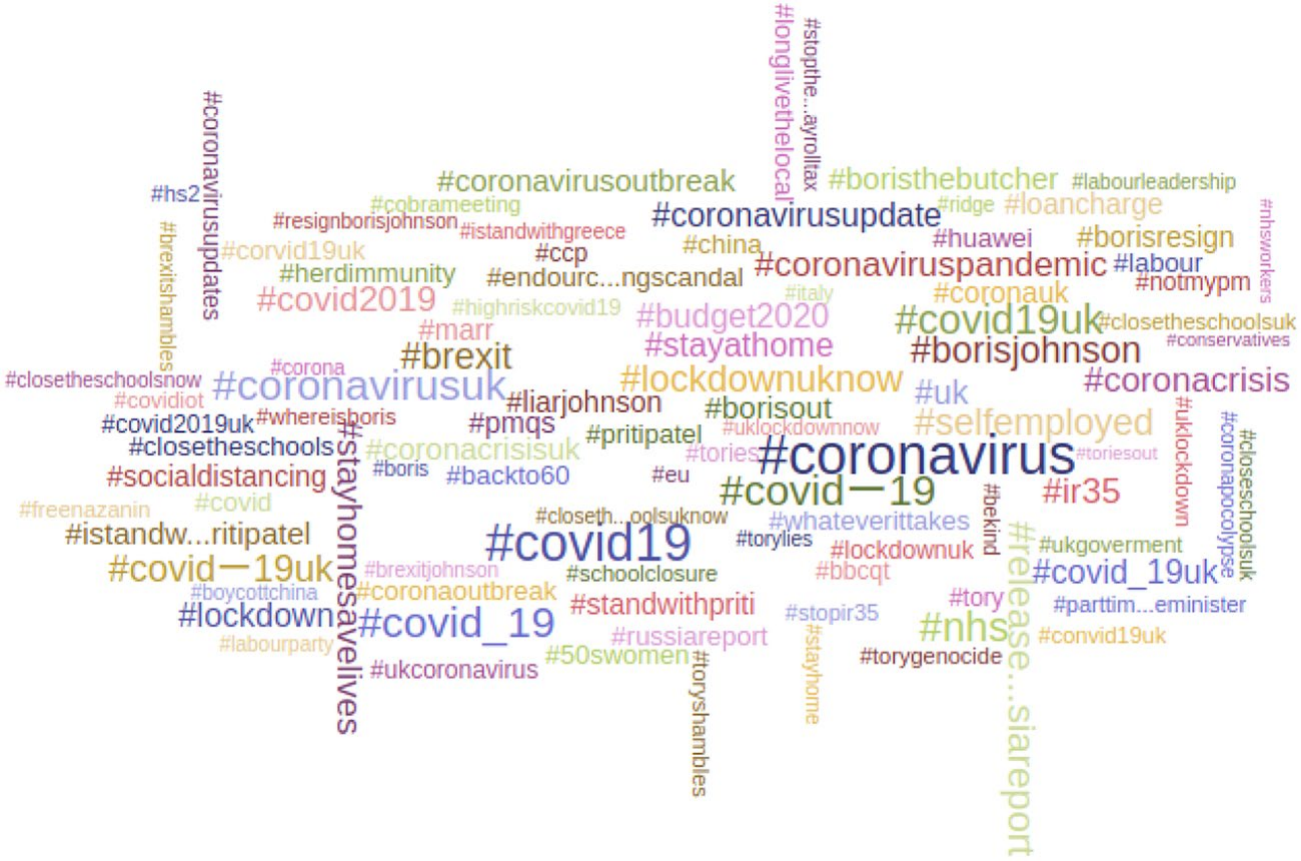
Top 100 hashtags in all replies sent to MPs—March 1st–22nd 2020 inclusive

The word cloud in Fig. 5 shows all hashtags in tweets to MPs in earlier part of March, and unsurprisingly shows a complete topic shift, to the subject of the epidemic, to the virtual exclusion of all else.

**Table 5:** MPs with greatest number of replies from March 1st to March 22nd 2020 inclusive

Name	Authored	replyTo	COV	Abusive	% Ab	% COV	% Total COV
**Boris Johnson**	45	160,356	28,818	7684	4.792	17.971	49.380
*David Lammy*	120	43,386	1602	2952	6.804	3.692	2.745
**Matthew Hancock**	100	42,520	7167	1800	4.233	16.856	12.281
*Jeremy Corbyn*	44	42,435	1824	3244	7.645	4.298	3.125
**Rishi Sunak**	47	25,534	2225	284	1.112	8.714	3.813
**Nadine Dorries**	52	24,731	1275	466	1.884	5.155	2.185
*Jess Phillips*	154	20,931	394	541	2.585	1.882	0.675
*Lisa Nandy*	74	20,355	234	925	4.544	1.150	0.401
*Richard Burgon*	145	20,281	446	1222	6.025	2.199	0.764
*Zarah Sultana*	107	18,796	204	815	4.336	1.085	0.350

Cell indicates party membership; bold for conservative, italic for Labour

We see from Table 5 that with the arrival of COVID-19 in the UK, Health secretary Matt Hancock became more prominent on Twitter at this time, though attention was not more abusive. Attention on chancellor Rishi Sunak also increased and was not abusive. This is consistent with previous research that indicates a public willingness and desire to trust authorities in a crisis [46, 67]. We see a high level of attention on Boris Johnson, but the abuse level is lower than was seen for him in previous years (we found 8.39% in the first half of 2019 as mentioned above; in 2017 as foreign secretary Mr. Johnson received similarly high abuse levels in high volumes). Negative attention on Labour politicians is high, but note that this was also the case before the start of the epidemic in the UK.

Matt Hancock received 11% abusive replies (3% of all abuse to MPs in the March pre-lockdown period) to the following tweet, in which he released his Telegraph article on the government’s response to the virus.

https://twitter.com/MattHancock/status/1238960146342084609.

NEWS: My Telegraph article on the next stage of our #coronavirus plan: < *link*>

We must all do everything in our power to protect lives

Critics were angry that Mr. Hancock would post important government information, during a time of extreme uncertainty, behind a pay wall. This goes back to the public’s information-seeking needs during a health crisis [46, 67]. Not only is information important for collective sense-making, it is also important for determining personal risk [70].

#### Beginning of lockdown

With the commencement of lockdown, the rise of the hashtags “#stayhomesavelives” and #lockdownuknow shows a shift toward comment on the practical details. Support for the lockdown appears to be high at this stage, with the top ten hashtags featuring only pro-lockdown or generic COVID-19 tags. Attention continues to focus on Boris Johnson (see Gorrell et al. [33] for complete word clouds and tables as well as histograms for each period), and is even less abusive than previously, largely due to a surge in non-abusive attention in conjunction with his being diagnosed with COVID-19. By volume, the most abuse-generating tweet was Boris Johnson’s illness announcement, but as a percentage, this was remarkably un-abusive, as discussed above, with only 2.3% abuse. The high abuse count follows only from the very high level of attention this tweet drew.

Moving into April 2020, the rise of the hashtag “#newstarterfurlough” shows that, prior to Donald Trump’s “liberation” tweets and the visible emergence of an anti-lockdown backlash, attention has already begun to focus on the economic cost of the lockdown, as illustrated by the prominence of hashtags such as #newstarterfurlough and #wearethetaxpayers.

Boris Johnson’s abuse level continues to be low as his illness takes a serious turn. In the context of the pandemic, different influences from the public also have a measure of authority. For example, the high abuse level toward Jack Lopresti during this period relates to his controversial opinion that churches should open for Easter. Strong opinion in conjunction with a religious event is part of a pattern that we see in conjunction with Eid in the next section.

https://twitter.com/JackLopresti/status/1247508135029411841 (18% abuse, 6% of all abuse sent to MPs between April 1st and 16th inclusive):

Open the churches for Easter – and give people hope https://telegraph.co.uk/news/2020/04/06/open-churches-easter-give-people-hope/?WT.mc_id=tmg_share_tw via @telegraphnews

https://twitter.com/JackLopresti/status/1247894726486798342 (17% abuse, 3% of all abuse sent to MPs between April 1st and 16th inclusive):

Today I wrote to The Secretary of State @mhclg and also sent a copy of this letter to Secretary of State @DCMS to ask the Government to consider opening church doors on Easter Sunday for private prayer.

#### Lockdown backlash

From mid-April 2020, a notable backlash against lockdown began to emerge. Hashtags now appear to be critical, often economically focused but also including accusations of lying against China, Boris Johnson and Conservatives, and references to the shortage of personal protective equipment for medical workers. The distinct change in tone echoes events in the USA.^19^

In this context, it is interesting, therefore, that the tweet receiving the most abusive response by volume (it also received a striking level of abuse by percentage) is this one by Ed Davey.

https://twitter.com/EdwardJDavey/status/1253882262715842560 (19% abuse, 5% of all abuse toward MPs for the period):

A pre-dawn meal today

Preparing for my first ever fast in the holy month of Ramadan

For Muslims doing Ramadan in isolation, you are not alone!

#RamadanMubarak

#LibDemIftar

The following tweet also attracted high levels of abuse by volume:

https://twitter.com/jeremycorbyn/status/1253341601599852544 :

Ramadan Mubarak to all Muslims in Islington North, all across the UK and all over the world.

This tweet received 11% abusive replies, 2% of abuse for the period—this was also St George’s Day, so perceived as evidence of anti-English sentiment, as in the following paraphrased replies for example: “@jeremycorbyn So nothing about St George’s day then? Ah, that’s because we are English, the country you wanted to run but hate with a vengeance. And you wonder why you suffered such a huge defeat at the election” and “@jeremycorbyn So no mention of St. George’s day then? You utter cretin.”

These attempts to reach voters and how they are perceived by those not within the same ideological framework will be discussed in “Ideology”.

#### Lifting of restrictions

As lockdown begins to be eased in May 2020, we see a return to a high level of focus on Boris Johnson, with 194,000 replies compared to Matt Hancock’s 124,000 as the next most replied to MP. Other senior conservatives are also prominent. High levels of abuse are received by ministers who defended Dominic Cummings’ actions on Twitter; Matthew Hancock (6.37%), Oliver Dowden (8.17%), and Michael Gove (7.13%). Boris Johnson also receives more abuse than he did in the previous period (4.92%). Example tweets are given below of ministers defending Mr. Cummings:^20^

https://twitter.com/MattHancock/status/1264162359733555202 (7% abuse, 7% of abuse for the period):

I know how ill coronavirus makes you. It was entirely right for Dom Cummings to find childcare for his toddler, when both he and his wife were getting ill.

https://twitter.com/MattHancock/status/1264975804208947208 (9% abuse, 4% of abuse for the period):

Dom Cummings was right today to set out in full detail how he made his decisions in very difficult circumstances. Now we must move on, fight this dreadful disease and get our country back on her feet

Hashtags show a high degree of negative attention focused on the partial treatment of Dominic Cummings, while continued attention on the economic plight of new starters is also in evidence. This signals the beginnings of contention that will blossom in the periods after this study was concluded.

### Ideology

The ideologies that influenced Brexit, such as anti-prejudice and tolerance [1], are still apparent in the context of COVID-19. “Virtue signalling” is a common complaint attached to nearly every tweet that addresses issues of inequality and some that are trying to engage voters (see “Findings: social media activities during COVID-19 (RQ3)”). Virtue signalling is defined as behaviour that indicates support for causes or sentiments that carry moral value, such as donating to charity [73], without much actual effort or care for the topic behind it. Disagreement on what constitutes virtue signalling versus actually caring about a social issue creates a “liminal hotspot” where misunderstanding takes place [1]. We go more deeply into the subject of virtue signalling in “Social meedia activities: subjectivity in escalation and virtue”.

In many cases, identity and ideology are interrelated through experience. Individuals from minority backgrounds speak about racism more often, also in context of COVID-19, potentially because they experience it. When MPs from under-valued or under-represented minorities speak to their voters about racism, they are not only speaking to voters who need to understand experiences of racism, but also to voters who experience it directly. If the MP has a track record in working toward racial justice, their election is a signal that they should keep doing this work. Of the 15 tweets shared by Women of Colour in our qualitative sample (190 Tweets receiving a high percentage and number of abusive replies), more than 50% are about engaging voters and being proactive toward issues of inequality (we see this in more detail in Contentious issues: disrupting the mainstream discourse and in Table 6). Another 40% are direct rebukes of authority from women in opposition parties. It seems that women of colour are disproportionately carrying the flame for the highly abused topic of inequality, as we see in Fig. 9. This may have partly to do with the party they belong to, but it appears to be also partly about the topics and expertise these women bring to the table. For example, Bell Ribeiro-Addy was elected for the first time in December 2019 after a long career of addressing inequality in migration. She addresses Sinophobia in communications about coronavirus and its origins in this tweet:

https://twitter.com/BellRibeiroAddy/status/1247570145733758980

As senior Conservatives publish Sinophobic screeds in the rw press to distract from their own Government’s lethal complacency, it’s clear racism won’t stop for #Coronavirus. Neither must we opposing it. Join the fightback in an hour! http://ow.ly/4GBm30qw1jX

This quote communicates proactiveness, for example, with the words “oppose” or “fightback”. As such, this is the kind of statement that is not meant for those who do not accept racism as a fact of experience, or who do not think it is an important issue in the context of COVID-19. It is a call to action for those who do.

This tweet, from MP Rupa Huq, addresses the lack of women ministers present at press briefings:

https://twitter.com/RupaHuq/status/1246107696652320769

Once again headed up by a man for the umpteenth time - who we only heard a week ago had tested #COVID19 positive to boot. When will Downing Street allow a woman minister to front up one of these press shindigs?

Several responses to this tweet ask why the MP chooses to focus on gender, given that the roles most relevant during the COVID-19 pandemic just happen to be held by men. Or, they ask her to explain why she believes a woman could do a better job. Some responses also refer to this as “virtue signalling”, though the MP is a woman and her comment is about representation of women.

In terms of the General Elections in 2015 and 2017, topics of concern were primarily around the economy, Europe, and the NHS. While Europe and Brexit are still present as important subjects, our topic analysis and the hashtags collected in each period show that the economy is now the greatest concern for users on Twitter, along with various implications for public health, survival of businesses, unemployment, and social welfare. The significant financial support that the government has provided during COVID-19 has revived discussions about socialism and capitalism more generally as economic models:

https://twitter.com/jeremycorbyn/status/1238897340309790721

There’s no statutory sick pay for part-time, low-paid or zero-hours contract workers. And the rate of sick pay isn’t enough to live on. Wrong at any time - but dangerous while people who might be ill are asked to stay home. The system is broken and now is the time to fix it.

This quote from Jeremy Corbyn attracted supportive messages, including some that are abusive toward Boris Johnson or other members of the Conservative party. However, there were also many criticisms of the tweet, which primarily express exasperation with Mr. Corbyn or chastise him for bringing this subject up in the middle of a crisis. If one agrees with Mr. Corbyn and feels that this crisis has only further exemplified the failings of the social welfare system in the United Kingdom, his words will resonate. If not, they will most likely aggravate.

**Table 6:** Controversial topics that are expressed MPs’ posts on Twitter, to which the MP received abusive replies

Controversial topic	No. tweets	Description	Examples
People & Communication Style	47	Tweets about specific people and their actions or the way that they communicate	*Dom Cummings followed the guidelines and looked after his family. End of story.*- Oliver Dowden
Covid Response & Impact	67	Tweets about the COVID-19 pandemic and any responses or impacts	*Latest NHS advice: If you have: a new continuous cough OR a high temperature You should stay at home for 7 days. Read the full guidance now:* http://NHS.uk/coronavirus - Boris Johnson
Inequality	45	Tweets about inequality in any form, be it racial, gender specific, religious, class-based, etc.	*Amazing. Dave telling it like it is.* - John McDonnell
Brexit	14	Tweets about Brexit	*Good to hear EU Commission saying they are fine with an Australia style deal or a bespoke Free Trade Agreement. So let’s get on with it. No need to argue over it all year.* - John Redwood
Home rule & nationalism	17	Tweets that appear to address long-standing conflicts about the Union and its Governance, including pro-Scotland and anti-SNP sentiment	*Really the most politically frustrating aspect of this crisis is not having an independent Scotland to do the emergency guaranteed income, to do the testing - to take a different path. We are instead shackled to inept economic extremists at Westminster.* - Angus MacNeil
Grand total	190		

Descriptions and examples included

### Affect

Previous work has suggested that impassioned speech provides a measure of credibility on its own [1]. MPs have been asked to consider the tone of their messages to the public and to one another in Parliament [20]. However, in COVID-19, in which the country is embroiled in a large public health crisis, it is difficult to determine exactly how tone impacts the political discourse. When we were looking for escalations in our qualitative sample, we looked at critiques of MPs’ tweets to try and understand what someone might object to in a given statement. Typically, hyperbolic language, sarcasm, insult, and making something personal are the escalations that are named. However, to avoid potentially classifying a tweet as escalating for using strong language around events that are urgent matters of public health, we required at least two measures of escalation to be present to classify a tweet as a potential escalation. When examining our sample, escalations were present in about a quarter of the tweets.

As a percentage of replies, a notable tweet was the following:

https://twitter.com/HenrySmithUK/status/1263394101002674176 (13% abuse, 2% of abuse for the period of May):

Not that I should be surprised by the lazy left but interesting how work-shy socialist and nationalist MPs tried to keep the remote Parliament going beyond 2 June.

In the context of an increasingly uncertain economic situation for many individuals, respondents felt that Mr. Smith was accusing those who have been furloughed or who are shielding of avoiding work. Several respondents also implied that work in communities to provide social support was not being valued. Though Mr. Smith’s comments (and his affect) may have been directed at his colleagues in Parliament, he hits a mark with left-leaning members of the British public, as well.

Many other tweets ($n=60$) are about rebuking authorities, which is sometimes done using strong language. We have 14 tweets from David Lammy in the sample, which is more than 7% of the sample. Many of these tweets include some sort of escalation indicator, such as hyperbole, sarcasm, or personal insults that critics tend to pick up on in their replies (see example in Table 2. Once again, this definition of “escalation” was defined by those who criticised the tweets. To understand this further, we extended our analysis to look more deeply at the specific subject matter being discussed. The subjects which Mr. Lammy is discussing are urgent and controversial in British political discourse, such as racism, Brexit, and more personally, the leadership of Boris Johnson. It is not clear whether or not he is targeted because of his communication style, the topics he discusses, his party membership, or because of his ethnic background.

Affect and race are connected in how much anger and frustration those from a minority background are expected to express by a predominantly White society about issues that White people may find largely irrelevant [2]. The stereotypes of the “angry Black woman” (or man) persist, in particular with connection to the topic of racism or inequality more generally.

## Findings: social media activities during COVID-19 (RQ3)

Our third research question asked: which social media activities of UK MPs during the COVID-19 pandemic receive the most abusive replies? To answer this question, we applied our coding scheme described in 4.3 to a sample of tweets that received a substantial number of replies that contained abusive language (see “Introduction”). The purpose of this was to identify qualitative differences in authors, content, or delivery that may help to explain the negative discourse related to their tweets and highlight any other social factors at play. In total, we identified 190 tweets meeting these criteria.

66 MPs authored the 190 tweets that received the highest number and percentages of abusive replies. 17 MPs are women, which is approximately 35% of the sample. Women make up approximately 30% of UK Parliament.^21^ However, of the 17 women, 41% are women of colour ($n=7$), though women of colour only make up a small percentage of an already small percentage MPs from a “minority” background.^22^ It is important to note that none of these women are members of the conservative party. In fact, Labour politicians have authored 108 of the 190 tweets in this sample. Conservatives authored 53, Liberal Democrats 16, the Scottish National Party 11, and the Democratic Unionists 2. To break this down further, we had 4 conservative MPs who are female (all white), and 25 male MPs. For the Labour Party, that split is more even, with 11 women and 14 men. Table 7 gives corpus statistics in terms of tweets authored. “Tweets” is number of tweets authored, “% of corp” is the percentage of the qualitative corpus that number constitutes, and “% Repr.” is the representation that demographic has among MPs with Twitter accounts for comparison. “# Replies” is the number of replies tweets by that demographic in the qualitative corpus received, and “# Abusive” is the number of those replies that were abusive (recall that the tweet is only included if it receives a high level of abuse).

**Table 7:** Proportion of the qualitative corpus (most abused tweets) authored by different demographics, alongside representation of that demographic among MPs on Twitter for comparison, the number of replies, and the number of abusive replies received by those tweets

Demographic	Tweets	% of corp	% Repr.	# Replies	# Abusive
White men	348	72.63	60.63	257,960	28,263
Men of colour	25	10.53	4.36	28,727	2938
Women of colour	32	7.89	5.57	30,999	3198
White women	169	8.95	29.44	33,435	3391

### Social media activities: subjectivity in escalation and virtue

Examining each tweet, we had 11 categories of social media activities, plus one additional “unclear” media category (see Table 2). We added a modifier to the media activity of “escalation”, if there were combinations of what we referred to as the five indicators of escalation: the presence of hyperbole (language that is perceived as having high valence), sarcasm or flippancy, insult, and abusive language, making something personal (criticising the individual rather than their actions) or solidifying “us and them” narratives. These escalation indicators were derived from how critics and abusers of the tweets in our sample speak about escalating language and what they think is antagonistic. If a tweet only contained escalation indicators and no other content, it was categorised as “escalation” only. Examples of each category are provided in Table 2 and the full qualitative sample and coding notes are provided at the URL given in “Availability of data and material”.

Escalations were particularly subjective and, therefore, difficult to classify. However, only 22 tweets contained some measure of escalation and 9 were coded as “Escalation” only. Here is an example of a tweet from Labour Party member Ian Lavery that contains an escalation, in addition to its main media activity of rebuking authorities:

https://twitter.com/IanLaveryMP/status/1238569895618625536

So does this “herd immunity” @BorisJohnson strategy mean accepting the end of life for many elderly & vulnerable people But others should be fine ? Just asking for the elderly lady across the street.

This tweet was categorised as an escalation, because it contains both sarcasm and hyperbole.

Some tweets received criticisms and abuse related to a particular event or pattern of behaviour. We have 12 examples of this in our sample. Examples of this include Dominic Cummings’ behaviour during lockdown in May (see “Events”), the birth of Boris Johnson’s child, or Sammy Wilson’s previous voting record on the NHS (when combined with a tweet promoting clapping for the NHS).

**Fig. 6:**
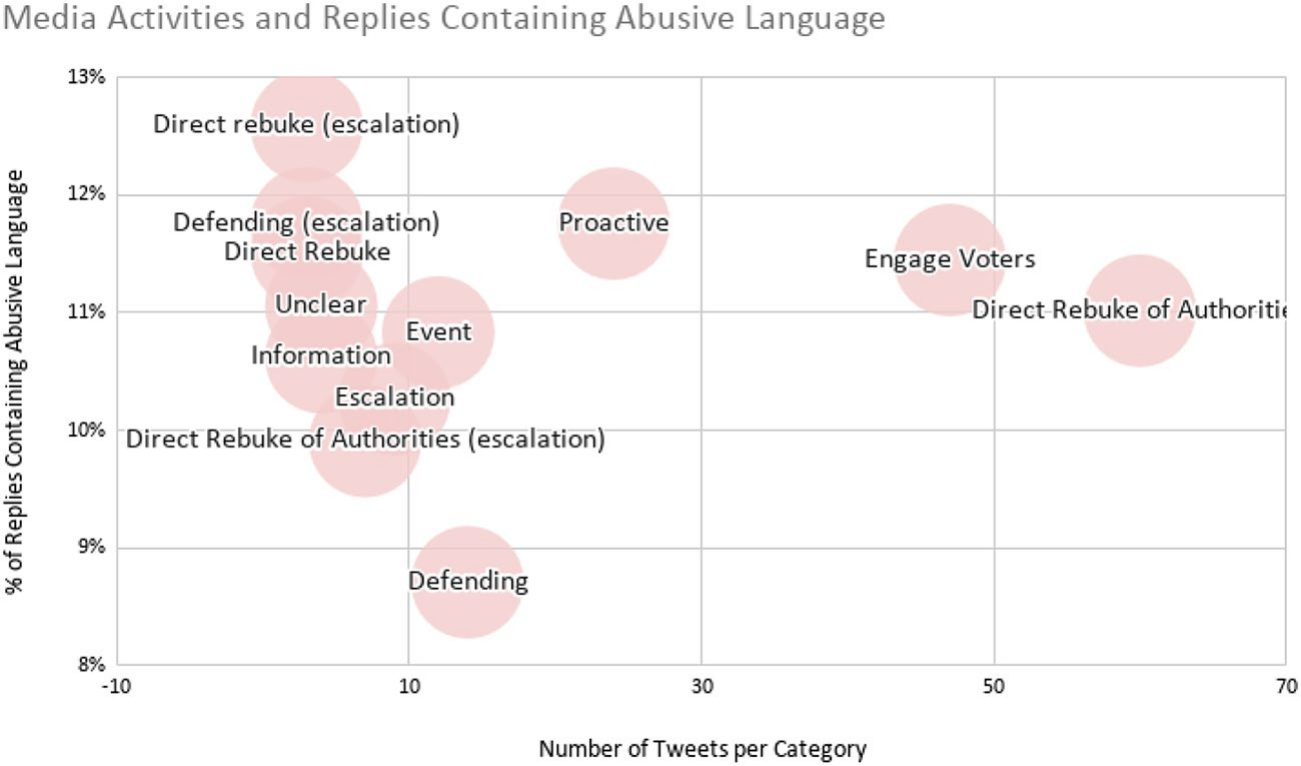
Media activities and replies containing abusive language

Figure 6 shows the media activity category and the number of abusive replies per category. While tweets with escalations may attract the higher percentages of abusive replies, the most common activities getting abusive replies are those ordinary to the job of an MP. The most common media activities receiving replies that contain abusive language are Direct Rebuke of Authorities ($n=60$) and Engaging Voters ($n=47$). The following quote from Lisa Nandy (coded as a Direct Rebuke of Authorities) received 9% replies including abusive language:

https://twitter.com/lisanandy/status/1237339808017547264

It is irresponsible and short-sighted from the government to rule out extending the post-Brexit transition period. We should be taking action now to provide certainty for business in the face of this global economic challenge.

This is a fairly standard argument from a member of the opposition party who was a “Remain” voter, preferring a “soft Brexit”.^23^ Likewise, the following tweet from conservative Jack Lopresti is an attempt to speak to a core group voters and champion their interests:

https://twitter.com/JackLopresti/status/1248306181522763778

If off-licences and takeawayys are open, churches should be, Tory MP claims https://t.co/aA2CY06XrU

This tweet was sign-posting to an article in the Telegraph in which Mr. Lopresti makes his views known. This tweet attracted nearly 12% abusive replies.

Perhaps unsurprisingly, all of the parties receive replies that contain abusive language when they do the parts of their job that aggravate the other parties. For example, the parties in opposition criticise the party in power, which will defend itself. All parties attempt to reach voters. However, the left and left-centrist parties tend to reach out to voters that conservatives do not, such as religious minorities, migrants, and People of Colour. The subject of “virtue signalling” arises in criticisms of this type of social media activity. This is evident in the large amount of abusive replies received by Liberal Democrats in response to their participation in Ramadan (see “Events”).

As mentioned previously, virtue signalling is defined as communicating support for a specific issue with high moral value (such as fighting racism) without providing tangible support and effort [73]. When the accusation of virtue signalling is levelled, the implicit assumption is that the gesture is empty or amounts to “moral grandstanding” [64] without substance behind it. In this case, in the past 10 years, evidence shows that the Muslim community has shifted support from the Labour Party to the Liberal Democrats.^24^ The party has responded to this, attempting repeatedly to elect a Muslim MP. The party has shown some attention to the social challenges of this group, suspending a candidate in 2019 for his comments online doubting the existence of Islamaphobia.^25^ They have also had a few gaffes, showing a lack of awareness of the culture,^26^ and have still not had a Muslim MP, despite efforts.^27^ Still, there is also evidence that the gesture of fasting and donating to charity as part of Ramadan was perceived as showing solidarity with the Muslim community during the COVID-19 pandemic.^28^ Virtue signalling needs to be considered within a framework of whose attention is being courted and whether or not that community views the attention as tokenistic or meaningful.

### COVID-19 topics: priorities and leadership

Our second round of coding dealt with the COVID-19 subject referenced or alluded to in the tweet, which we determined inductively by going through the set of tweets using thematic analysis. We assigned tweets to one of eight categories, including one “non-COVID” category ($n = 37$), if the topic was not related to COVID-19. These non-COVID topics include the floods that happened just prior to the pandemic, some more general thoughts on platform issues that are continuously relevant, such as budget and migration. Many non-COVID- related tweets were posted before COVID-19 had reached the UK to such a significant extent. Topics include the cabinet reshuffle, class issues, Brexit, and migration.

**Fig. 7:**
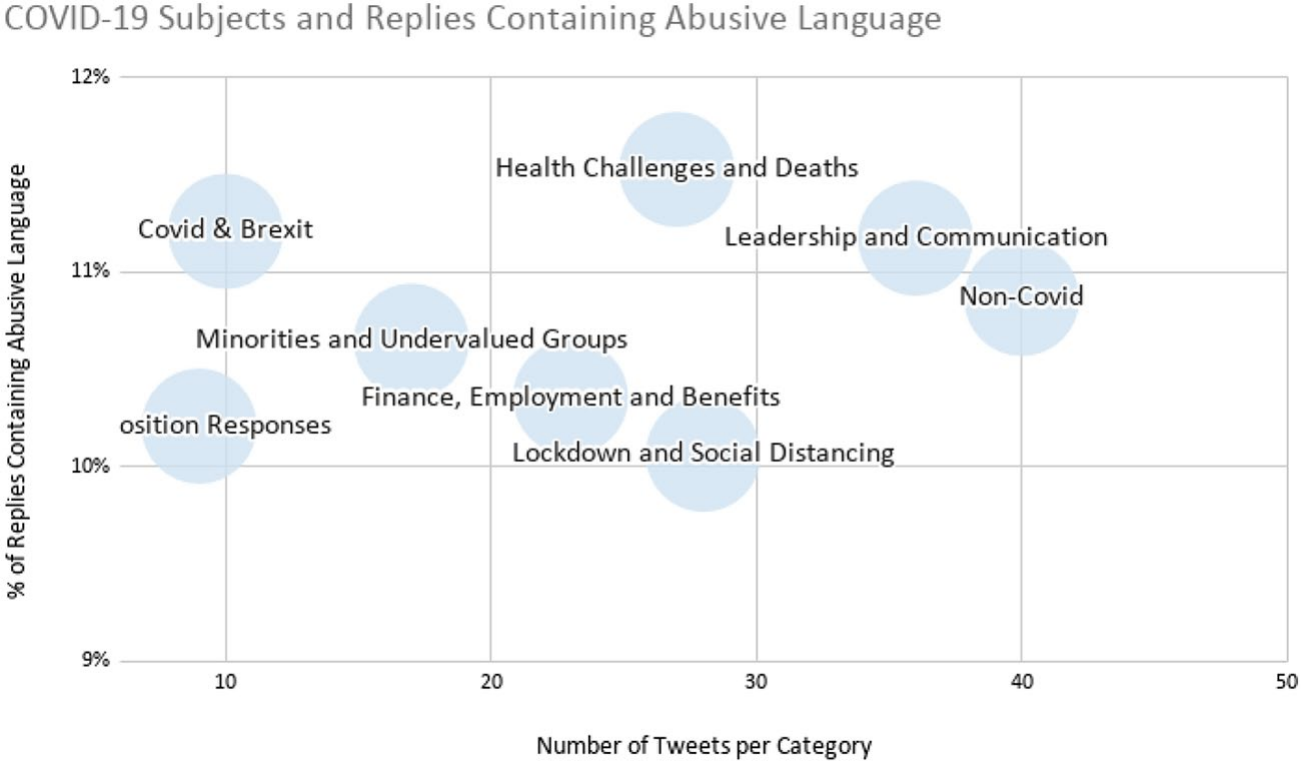
COVID-19 subjects and replies containing abusive language

After COVID-19 began to take hold, those non-COVID topics shift and are primarily related to specific issues or events, such as Jeremy Corbyn stepping down and Keir Starmer taking lead of the Labour Party, renewed references to a One UK policy/One Parliament, Boris Johnson and the birth of his child, and the Liberal Democrats celebration of Ramadan. Within the COVID-19 topics, some more specific categories, such as “fatalities” or the NHS, were absorbed under the main category of Health challenges and deaths to arrive at groupings that including roughly the same number of examples from our data sample. The full list of categories can be found at the URL provided in “Availability of data and material”. In Fig. 7, however, we show which topics around COVID-19 were associated with receiving more replies that are abusive in our qualitative sample (Table 8).

**Table 8:** COVID-19 topics that are expressed MPs’ posts on Twitter, to which the MP received abusive replies

COVID-19 topic	No. tweets	Description	Examples
Covid & Brexit	10	Discussing the impact of COVID-19 on Brexit	*The country cannot afford the economic damage and chaos of a no-deal Brexit at the end of this year. Time is running out for the Government to agree to an extension and people’s livelihoods first.*- Layla Moran
Health challenges & deaths (e)	27	Discussion of and reporting about fatalities during COVID-19	*Italy: Population - 60 m Coronavirus cases - 21,157 Deaths - 1,441 UK: Population - 66 m Coronavirus cases - 1,372 Deaths - 35 Italy had their first confirmed case 24hrs before the UK. The strategy is working.* - Rob Roberts
Finance % benefits	23	Discussion of how financial support and benefits will work during COVID-19. This includes discussion of what COVID-19 has shown us about our current economic models	*There is no statutory sick pay for part-time, low-paid or zero-hours contract workers. And the rate of sick pay is not enough to live on. Wrong at any time - but dangerous while people who might be ill are asked to stay home. The system is broken and now is the time to fix it.* - Jeremy Corbyn
Opposition responses	9	Defense or rebuke of Labour MPs specifically	*Intentionally misleading reporting is really disappointing at a time like this. I’ve spoken about the opportunity for people to get out there and help their local communities and those in need. Nonsense to suggest otherwise. We all need to do our bit to get through this crisis.* - Ian Lavery
Leadership & communication	36	Discussion of how policy or commentary has been delivered during the COVID-19 crisis	*The @theSNP continue to let down thousands of homes in rural and remote communities. We need an audit on how they’re spending broadband rollout money! See my exchange with the Government* - Jamie Stone
Lockdown & social distancing	28	Discussion of how lockdown or social distancing is impacting people, the economy and the virus	*I’m all in favour of wearing the appropriate face mask! @YesBikers* - Stewart Hosie
Minorities & Under-valued Groups	17	Any discussion about a group that is viewed as a minority group in the UK	*As senior Conservatives publish Sinophobic screeds in the rw press to distract from their own Government’s lethal complacency, it’s clear racism won’t stop for #Coronavirus. Neither must we opposing it. Join the fightback in an hour!* http://ow.ly/4GBm30qw1jX - Bell Ribeiro-Addy
Non-COVID	40	Any discussion about topics that are not linked to Covid-19 in direct ways, such as flooding	*A pre-dawn meal today Preparing for my first ever fast in the holy month of Ramadan For Muslims doing Ramadan in isolation, you are not alone! #RamadanMubarak #LibDemIftar* - Ed Davey
Grand total	190		

Descriptions and examples included

Leadership and communication ($n=39$), along with Lockdown and Social Distancing issues ($n=28$) were the COVID-19 topics that had the best representation in the sample, with high number of replies that contain abusive language. These two categories include issues such as perceived government inaction and tone (Leadership and Communication), and guidance or impacts around lockdown or social distancing, such as wearing a face mask (Lockdown and Social Distancing).

The following tweet from Labour MP Yvette Cooper attracted more than 8% abusive replies, addressing perceived confusion around guidance from the UK government:

https://twitter.com/YvetteCooperMP/status/1241849797155393537.

I watched the Prime Minister’s press conference in despair. In a public health emergency communication and information saves lives. Yet time & again the Government keeps failing to push out a strong clear message to everyone. For all our sakes they urgently need to get a grip.

The following tweet from Jacob Rees-Mogg linked to an article about the Queen isolating herself amidst COVID-19 concerns. It attracted 11% abusive replies, mostly for invoking the name of the Queen or comparing her experience to those of citizens who are struggling to meet their needs.

https://twitter.com/Jacob_Rees_Mogg/status/1239949070426419200.

As always an example to the nation: God save the Queen

https://t.co/N7egXeDzXD?amp=1

Discussion of Health Challenges and Deaths ($n = 27$) received the greatest percentage of abusive replies. This includes conversations around UK fatalities in comparison to other nations, support for the NHS, and issues with testing. This type of comparison, as mentioned previously (especially from someone in a left-orientated party), is generally received as negative, and even unpatriotic in some critiques.

Women of colour tended to discuss topics that address the needs of minorities and under-valued groups during COVID-19. White women have a profile more similar to men, in which questioning leadership and communication tended to be the COVID-19 subject for which they received more replies containing abusive language. Clearly, questioning the government in power may lead to criticism. Our literature review indicated that people tend to want to trust authorities during a crisis, though, perhaps, this will shift as the pandemic progresses. Labour politicians’ desire to keep the subject of racism and discrimination during COVID-19 at the forefront attracts some abusive comments for what is called “playing politics”, delivering “low blows” or “playing the race card” to the party in power. Naturally, the opposition parties believe that it is their job to rebuke authorities and suggest alternative policies. Likewise, racism is not seen as a platform issue, but a social issue that is continuously relevant. In this sense, what is relevant to COVID-19 and should be prioritised is being negotiated in some of this dialogue.

### Contentious issues: disrupting the mainstream discourse

**Fig. 8:**
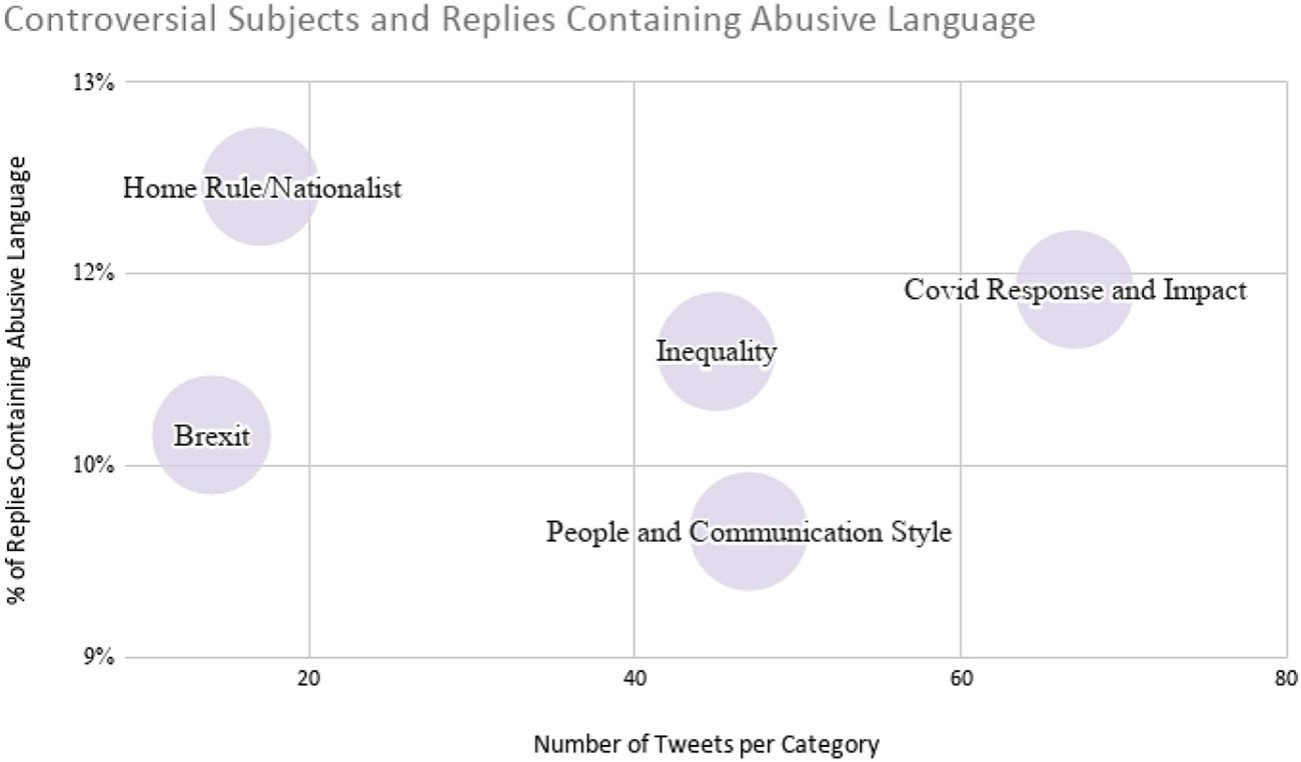
Contentious issues and replies containing abusive language

**Fig. 9:**
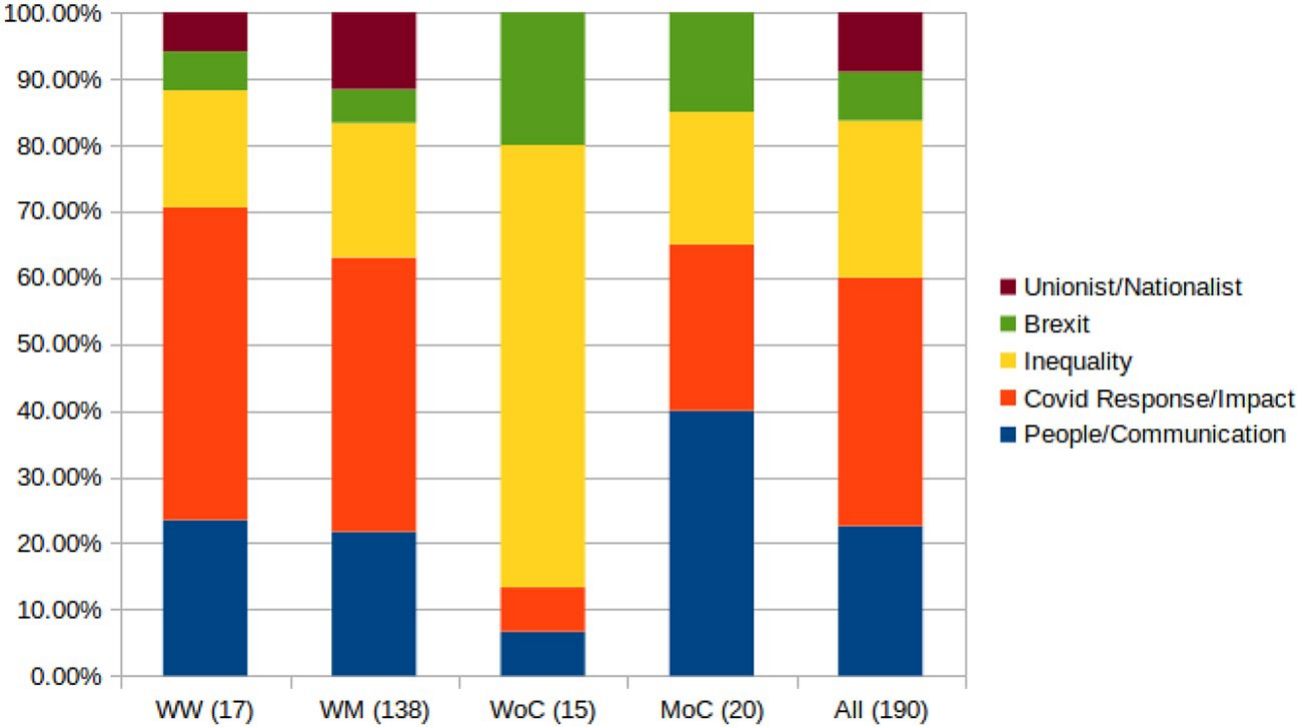
The 190 highly abused tweets in the qualitative sample, split by topic and by demographic. “WW” means white women, “WoC” means women of colour, and similarly for men

When we looked deeper at the controversy that might be latent in the topics above, we identified more than 50 distinct subjects from our open coding, and had one category of “unclear”. Some categories were related, such as Islam and Muslims, Racism, and Immigration. We reduced the categories to five predominant issues (see Fig. 8): Home Rule/Nationalist perspectives, Inequality and perceptions of inequality, Brexit (a continued issue with new relevance), COVID-19 Response and Impact, and finally, People and Communication (which includes subjects like personal folly, tone, etc.). Proportions of each subject to appear in tweets from different demographic groups, as well as overall, are shown in Fig. 9.

For example, the #stayalert slogan of the conservative government received considerable criticism for being confusing and potentially working against the goal of encouraging citizens to simply stay home. Criticising the government’s efforts, MP Ian Blackford tweeted:

https://twitter.com/Ianblackford_MP/status/1259250871814295552.

“#stayalert. What kind of buffoon thinks of this kind of nonsense. It is an invisible threat. Staying alert is not the answer #StayHomeSaveLives is.”

This tweet received both a number of abusive replies to Mr. Blackford for critising the government’s attempts to resolve complex challenges during the pandemic, and a number of supportive replies (some of which are also abusive toward the government and certain ministers). This polarisation indicates a liminal hotspot around trusting and critiquing authorities in crisis.

In terms of party insights, the Liberal Democrats received a considerable amount of attention for speaking about Ramadan and the Muslim community (56% of their sample). The SNP, as one might expect, gathers some abusive replies when tweeting support for Scottish independence or for promoting Scottish excellence. Hannah Bardell received abusive and critical replies for posting the following tweet:

https://twitter.com/HannahB4LiviMP/status/1238750253182050310.

Once again the PM following Nicola Sturgeon’s lead

The tweet was accompanied by an article from the Guardian^29^ about the Prime Minister’s decision to ban mass gatherings.

Angus MacNeil was called “divisive” and “divisionist” for the following tweet in response to clapping for the NHS:

It is “NHS Scotland”

Likewise, making statements in favour of uniting the four UK countries, or making light of those countries’ prerogative to handle the pandemic differently, attracts strong criticism and abuse. For example, Stephen Crabb received several abusive replies for the following tweet:

Quite the change in rhetoric from the days when Welsh Government were encouraging people to come to Wales and drive their 4x4’s right onto the beaches.

Another liminal hotspot is around who is expected to speak about social issues of injustice, which commonly attract abusive responses. Women of colour were speaking mostly about inequality when they received replies containing abuse (10 Tweets, which is 66% of our sample of women of colour, and 5% of our total sample). Men of colour more closely resembled the subject attention of both White women and White men on the more general discussion of COVID-19 response and leadership through the crisis (For all men, issues with Leadership, or COVID-19 response made up 60% of all topics; for white women, more than 70%. 10% of the topics discussed by men in their sample are about controversial people specifically, such as Boris Johnson, Jeremy Corbyn, and Dominic Cummings).

## Discussion and future work

All activity online can be viewed as communication and persuasion—there are people on different sides of different issues, vying for the public attention. This can attract positive and negative responses. While our small qualitative sample may not generalise across all categories, overall, what our investigation indicates is that dimensions of political discourse are mediated by perceptions of power, potentially due to the uncertain situation created by COVID-19, and audience. In the following sections, we summarise our findings about the influence of ideology, political authority, and affect on how the words of MPs are communicated and interpreted by the public during the COVID-19 pandemic.

### Power, ideology, and virtue

Ideology and virtue are connected in our qualitative data to what kind of power the individual or party has (or is perceived to have). When parties on the political left speak directly with and from under-valued communities, this may be perceived as virtue signalling by the political right. Likewise, when conservatives show a lack of tact toward excluded communities, especially from a position of power, this is judged more harshly by the ideological left.

Similarly, when the party in power communicated policy about controversial issues in the name of the people, it received push-back from people who did not vote for that party. When the left attempted to meet voters by discussing issues (racism, migration), a portion of the public was defensive (and sometimes offensive). Looking at successful interventions by opposition parties in the government’s activities, for example, may constitute an interesting area for future exploration. More specifically, this research could help to answer questions about the origins of priorities and disagreements in partisan politics.

### Power, authority, and vindication

What the answers to our three research questions indicate is that it matters who is “in charge”, when looking at how the public and other ministers respond to the social media activities of UK MPs during COVID-19. The party in power (along with its members) will have more responsibility to the public for mastering tone and explaining their actions. Opposition parties will have more difficulty in a health crisis to not be perceived as unnecessarily antagonistic. In addition, we found that it matters who a person is and what they represent, whether or not an individual will be perceived as a trustworthy authority. Many tweets in our sample appeared to be speaking to core groups of voters and other parliamentarians. They are not necessarily an invitation for debate.

### Power, affect, and vitriol

In terms of affect, our data show that it matters what you say and how you say it, particularly in connection with priors. If Jeremy Corbyn posts about racism, and has been continuously in the news for not handling antisemitism in his party, he will get some angry replies. If Sammy Wilson voted against a pay-rise for nurses in 2017, and then posts a “clap for carers” post on Twitter, those who remember his prior voting record will be angry. The more affected a tweet is, the more this appears to aggravate.

Vitriol as a result of one’s previous political statements or actions is one side of the story. Hate is another. The issue of what is abusive versus what is hate speech needs to be disentangled from both abuse and from racism. Racism does not only involve hate speech. It also involves (a) expecting people of colour to champion racial equality, as the breakdown of topics indicates and (b) framing racism as a fringe issue. This is evidenced in our dataset. Abuse, though uncomfortable and uncivil, is a different type of speech whose study may be useful for any number of discussions, potentially on the subject of agonism or counter-speech. Agonism argues that the contestations of the time can be used to renew democracy and strengthen public discourse [47]. Promising work on recognising an highlighting counter-speech in online communication is already on the horizon [15, 50].

## Conclusion

In this paper, we explored how UK MPs contribute to the information and communication environment during COVID-19, and the abusive replies that they receive. Contextualising these activities in terms of what the public expect during a health crisis, how ministers typically use social media to communicate in crisis, and which mitigating features of either the person or context interfere in those activities, we were able to advance the conversation about online abuse toward some new directions, such as how to understand virtue signalling or what it means to play party politics. Building on previous studies of abusive language toward British MPs, we offered a large-scale, mixed-methods study of abusive and antagonistic responses to UK politicians during the COVID-19 pandemic from early February to late May 2020. We found that—similarly to other key moments in British contemporary politics—political ideology, authority, and affect have played a role in how MPs social media posts were received by the public. In the context of COVID-19, we found that pressing subjects, like financial support or unemployment, may attract high levels of engagement, but do not necessarily lead to abusive dialogue. As with earlier findings, prominence and event surges impact the amount of abusive replies MPs received. In addition, the topic of the tweet (in particular if it is divisive) and the individual bringing that topic into discussion (their gender, ethnicity or party, for example) impacted levels of abuse. Women of colour appear to bring the topic of inequality to the table and this attracts a variety of abuse. Other MPs may be discussing inequality and not receiving abuse (which this work did not cover).

In conclusion, this work contributes to the wider understanding of abusive language online, in particular that which is directed at public officials. Issues of power, which are crystallised in terms of political power or social power, impact communication at all stages.

## Data Availability

Data are available here: https://gate-socmedia.group.shef.ac.uk/abuse-mps-covid/.

## Footnotes

1 https://www.bbc.co.uk/news/election-2019-50246969.

2 https://ourworldindata.org/mortality-risk-covid.

3 https://www.pewresearch.org/internet/fact-sheet/social-media/.

4 https://www.gov.uk/government/publications/coronavirus-COVID-19-support-for-care-homes/coronavirus-COVID-19-care-home-support-package.

5 https://www.ukyouth.org/wp-content/uploads/2020/04/UK-Youth-COVID-19-Impact-Report-External-Final-08.04.20.pdf.

6 https://www.bbc.co.uk/news/uk-wales-52614204.

7 https://www.bbc.co.uk/news/uk-scotland-53195166.

8 https://www.bbc.co.uk/news/uk-northern-ireland-53386976.

9 https://www.theguardian.com/uk-news/2020/may/14/police-vow-to-break-up-planned-anti-lockdown-protests-in-uk-cities

10 https://www.telegraph.co.uk/news/2020/04/20/coronavirus-world-erupts-protest-against-lockdown-pictures/

11 https://www.washingtonpost.com/world/europe/face-masks-coronavirus-uk/2020/07/14/d05dfb7c-c5d4-11ea-a825-8722004e4150_story.html.

12 https://www.telegraph.co.uk/news/2020/04/06/britains-hubristic-scientific-advisers-wrong-public-should-wearing/.

13 https://www.theguardian.com/politics/2019/sep/30/westminster-parties-sign-pledge-to-use-moderate-language. https://www.bbc.co.uk/news/uk-politics-49833804. https://www.bbc.co.uk/news/uk-37978582.

14 http://www.legislation.gov.uk/ukpga/1986/64/contents.

15 http://moonshotcve.com/COVID-19-conspiracy-theories-hate-speech-twitter/.

16 https://www.southwalesargus.co.uk/news/18444799.wearing-face-covering-mask-wales-not-compulsory/.

17 https://www.forbes.com/sites/tarahaelle/2020/06/19/risking-their-lives-to-save-their-lives-why-public-health-experts-support-black-lives-matter-protests/#11b5ac96851b.

18 https://twitter.com/RichardBurgon/status/1247248198932062208.

19 e.g., https://www.theguardian.com/global/video/2020/apr/16/armed-protesters-demand-an-end-to-michigans-coronavirus-lockdown-orders-video.

20 More tweets by ministers defending Mr. Cummings: https://twitter.com/OliverDowden/status/1264221876374646786. https://twitter.com/michaelgove/status/1264126108733186050.

21 http://www.ukpolitical.info/female-members-of-parliament.htm.

22 https://theconversation.com/black-british-citizens-want-more-than-complacency-from-this-government-140297.

23 https://www.theyworkforyou.com/mp/24831/lisa_nandy/wigan/votes.

24 https://www.aljazeera.com/focus/britishelection/2010/05/20105312436485579.html.

25 https://metro.co.uk/2019/04/18/lib-dem-candidate-suspended-comments-muslims-9243430/.

26 https://www.telegraph.co.uk/politics/2020/04/26/lib-dem-councillor-apologises-tweeting-photo-bacon-solidarity/.

27 http://muslimnews.co.uk/newspaper/top-stories/record-18-muslim-mps-elected-majority-women/.

28 https://www.easterneye.biz/lib-dem-mps-to-fast-during-ramadan-to-show-unity-for-muslim-community/.

29 https://twitter.com/guardiannews/status/1238591827449651205/photo/1.
